# The Diagnostic Value of Pulsed Wave Tissue Doppler Imaging in Asymptomatic Beta- Thalassemia Major Children and Young Adults; Relation to Chemical Biomarkers of Left Ventricular Function and Iron Overload

**DOI:** 10.4084/MJHID.2015.051

**Published:** 2015-08-24

**Authors:** Seham M Ragab, Waleed M Fathy, Walaa FAbd El-Aziz, Rasha T Helal

**Affiliations:** 1 Department of Pediatrics, Faculty of Medicine, Menoufia University. Naser street, Shebeen El-koom, Menoufia, Egypt; 2 Department of Clinical pathology, Faculty of Medicine, Menoufia University. Naser street, Shebeen El-koom, Menoufia, Egypt; 3 Department of Cardiology, Faculty of Medicine, Menoufia University. Naser street, Shebeen El-koom, Menoufia, Egypt

## Abstract

**Background:**

Cardiac iron toxicity is the leading cause of death among β-halassaemia major (TM) patients. Once heart failure becomes overt, it is difficult to reverse.

**Objectives:**

To investigate non-overt cardiac dysfunctions in TM patients using pulsed wave Tissue Doppler Imaging (TD I) and its relation to iron overload and brain natriuretic peptide (BNP).

**Methods:**

Thorough clinical, conventional echo and pulsed wave TDI parameters were compared between asymptomatic 25 β-TM patients and 20 age and gender matched individuals. Serum ferritin and plasma BNP levels were assayed by ELISA.

**Results:**

TM patients had significant higher mitral inflow early diastolic (E) wave and non significant other conventional echo parameters. In the patient group, pulsed wave TDI revealed systolic dysfunctions, in the form of significant higher isovolumetric contraction time (ICT), and lower ejection time (E T), with diastolic dysfunction in the form of higher isovolumetric relaxation time (IRT), and lower mitral annulus early diastolic velocity E′ (12.07 ±2.06 vs 15.04±2.65, P= 0.003) compared to the controls. Plasma BNP was higher in patients compared to the controls. Plasma BNP and serum ferritin had a significant correlation with each other and with pulsed wave conventional and TDI indices of systolic and diastolic functions. Patients with E/E′ ≥ 8 had significant higher serum ferritin and plasma BNP levels compared to those with ratio < 8 without a difference in Hb levels.

**Conclusion:**

Pulsed wave TDI is an important diagnostic tool for latent cardiac dysfunction in iron-loaded TM patients and is related to iron overload and BNP.

## Introduction

Thalassemia is one of the most common genetic disorders. Thus, it is considered a global health problem. Worldwide, about 5% of the population carry globin variants. Beta (β)–Thalassemia is caused by the reduced synthesis of β-globin chains, which leaves an erythrocyte excess of unopposed α-chains resulting in ineffective erythropoiesis and chronic hemolytic anemia.^1^

According to the severity, β-thalassemias are classified into: transfusion dependent β thalassemias (TDT) or β-thalassemia major (TM), non transfusion dependent β-thalassemia (NTDT) or β-thalassemia intermedia (TI) and β-thalassemia trait (asymptomatic carriers). β–TM is the severest form that develops during the first year of life and requires lifelong transfusion therapy for survival.^2^

Although improving survival, repeated blood transfusion regimen causes iron overload and iron toxicity in different organs including the heart.^3^

Despite the progress in iron chelation therapy, congestive heart failure due to iron accumulation.

Is still the leading cause of death in β-TM patients.^4^,^5^

Iron overload in combination with other inflammatory and immunogenetic factors can cause left ventricular systolic dysfunction, dilatation and failure, whereas the sole iron overload may result in left ventricular diastolic dysfunction with myocardial restriction and subsequent pulmonary hypertension and right ventricular dilatation.^6^

Patients with TM may remain asymptomatic and global left ventricular (LV) function may be preserved until late in the disease process.^7^,^8^ So, early detection of myocardial dysfunction may be useful in the management plan.^9^

Echocardiography is an essential imaging modality for diagnosis of ventricular function,^10^ that allows exclusion of overt LV systolic dysfunction (left ventricular ejection fraction < 50%).^11^

However, changes of segmental wall motion – the early sign of myocardial dysfunction in thalassemia patients - may be subtle and could be missed by conventional echocardiographic examination which may remain normal until late stages during this disease process.^12^

Tissue Doppler Imaging (TDI) is a relatively new Doppler ultrasound modality that records regional systolic and diastolic velocities within the myocardium. It allows quantitative measurement of both systolic and diastolic velocities directly from the ventricular myocardium with the determination of the extent of mitral annular displacement in systole and diastole.^13^

This new technique can show additional information compared with other echocardiography techniques, detecting even minor changes before the occurrence of abnormal indices of global ventricular dysfunction.^14^

Brain natriuretic peptide (BNP) is one of the natriuretic peptide system that is stored in the myocardial cells as pre- proBNP. It is secreted from the heart as a result of direct wall stress, caused by either stretch or pressure affecting cardiocytes. Once released, BNP has pronounced natriuretic, diuretic and vasodilating properties, working to dramatically reduce volume overload and hypertension.^15^ BNP level is useful for the diagnosis of left ventricular systolic and diastolic dysfunctions and is correlated with the severity and prognosis.^16^,^17^

So, the aim of this work was to investigate the utility of pulsed wave DTI to detect latent or non-overt cardiac dysfunctions in asymptomatic TM patients and its relation to the iron overload assayed by serum ferritin and to BNP as a biomarker of cardiac dysfunction.

## Materials and Methods

This is a cross-sectional study, performed upon 45 subjects (patients and controls); 25 β-TM patients and 20 age and sex matched healthy individuals as controls.

The patient group included 25 multi-transfused β-TM patients (14 males and 11 females). Their ages ranged from 4 to 20 years with mean age of 12 ±5.79 years and median of 12 years. These patients were kept on a regular blood transfusion regimen (every 3–4 weeks) since infancy to maintain pre-transfusion Hb above 7 gm/dl and post-transfusion Hb above 10gm/dl. They were on long-term chelation therapy for at least one year either by Deferoxamine (DFO) monotherapy, 30–50 mg/kg body weight by subcutaneous infusion with an infusion pump for 8–12 h, 5 days per week (16/25=64%), oral Deferasirox monotherapy, 20–30 mg/kg/day, daily (4/25=16%) or combined therapy with DFO and Deferasirox (DFO at the dosage of 40 mg/kg/day for 3 days/week and daily Deferasirox at the dosage of 30 mg/Kg/day, 5/25=20%).

The study included cardiac asymptomatic TM patients with ejection fraction >55% and a normal resting 12-lead electrocardiogram (ECG).

Patients on cardiovascular treatment, with any cardiovascular complaints, documented arrhythmia, hypertension, renal disease, diabetes mellitus, congenital or rheumatic heart disease, use of medications altering myocardial functions or a history of smoking were excluded. Also, those who developed transfusion associated circulatory overload (TACO) or transfusion-related acute lung injury (TRALI) were excluded.

The control group consisted of 20 healthy age and gender-matched subjects (10 males and 10 females). Their ages ranged from 4 to 18 years with mean age of 10.9 ± 4.86 years and median of 10 years. They were free from acute (especially viral illness) or chronic illness (including cardiac diseases) with no family history of chronic hemolytic anemia. All controls had normal complete blood count (CBC), Hemoglobin (Hb) electrophoresis with normal ECG and conventional Echocardiographic findings. They had been randomly selected from children presented to our general outpatient clinic for routine check up especially for growth, or for non-specific complaints like non-specific abdominal pain.

This study had been carried out at the Hematology Unit, Pediatric Department in collaboration with Clinical Pathology and Cardiology Departments, Faculty of Medicine, Menoufia University, Egypt, in the period of time between January 2012 and September 2013. The study was approved by the ethical committee of Menoufia Medical School, and informed consent was obtained from the patient or his or her legal guardian.

### Methods

All participants in the study were subjected to a full history taking and comprehensive clinical examination including a cardiac examination.

For the patient group, a special emphasis was given to the age of the disease manifestations, time of the first blood transfusion, frequency of blood transfusion with calculation of red blood cells transfusion index (RBCsTI) during the last year, chelation therapy details, hepatic, renal, histories and history of splenectomy.

For all included children (patients and controls) weight and height were measured by the standard methods and plotted against age and sex specific centiles.

The participants were investigated by the following:

#### I- Conventional Echocardiography

Echocardiography, in the form of complete two-dimensional, continuous and pulsed wave echocardiographic examination, was done using ultrasound machine (vivid 9, General Electric Medical Systems, Horton, Norway), equipped with 5MHz variable frequency harmonic –phased array transducer with simultaneous ECG monitoring, performed without sedation, during normal respiration in the left lateral decubitus. Images were recorded in the standard parasternal long axis, apical four and two chamber views.

##### Conventional Echo-Doppler Measurements

Routine M-mode, two-dimensional continuous wave Doppler recordings were obtained for each subject. The left atrial (LA), aortic (AO) diameters and left ventricular ( LV) internal cavity dimensions including left ventricular end systolic diameter (LVESD) and left ventricular end diastolic diameter (LVEDD) were determined. LV ejection fraction (EF) and LV fractional shortening (FS) were measured using Teichholz’s M-mode formula.^18^

Transmittal flow patterns were obtained by pulsed-wave Doppler echocardiography from apical four-chamber view. The peak of early diastolic flow velocity (E), the peak of late diastolic flow velocity (A) and the ratio of E/A were measured.

#### II- Pulsed Wave Tissue Doppler Imaging (TDI) Measurements

The pulsed wave TDI was performed using the same machine. To display tissue velocities; from the apical 4 and 2-chamber views, the Doppler sample volume was placed at four different sites of the mitral annulus: anterior, lateral, septal and inferior walls in order to record major velocities. The following parameters were registered: mitral annulus systolic velocity (S′), mitral annulus early diastolic velocity (E′), mitral annulus late diastolic velocity (A′) and time intervals; isovolumetric contraction time (ICT), isovolumetric relaxation time (IRT) and ejection time (ET).

Then calculation of the mean E/E′(mitral inflow E wave/E′ mitral annulus velocity) ratio was done. According to the E/E′ ratio, patients were classified into: patients with E/E′ ≥15 (diastolic dysfunction), patients with E/E′ ≥ 8 but less than 15 (suspected diastolic dysfunction) and those with E/E′< 8 (without diastolic dysfunction).^19^,^20^

All pulsed-wave Doppler and PW-TDI parameters were measured at the end of expiration, at a sweep speed of 100 mm/s on three consecutive heart beats and the average for each was taken.

All data were obtained according to the recommendations of the American Society of Echocardiography.^21^

#### III-Laboratory investigations including

1. Complete blood count (CBC): using Beckman 750, Int, U.S.A, Auto-counter after calibration.
2. Serum ferritin level was measured by Enzyme Linked Immune Sorbent Assay (ELISA) technique (ELISA, GenWay Biotech, Inc, NP 000137, Swiss) on Microplate reader (Bio-Rad 680 Hercules, California, USA). The mean yearly serum ferritin level in the previous year was considered (on the average of 4 determinations) for patients and at the time of sampling for the controls.
3. Plasma BNP: Three ml venous blood samples were drawn by sterile vein-puncture on EDTA tube. Blood samples were immediately centrifuged for 15 minutes at 3000 rpm; plasma samples were separated then were stored at −20°C until analysis. Plasma BNP level was measured by ELISA using kits supplied by Ray Biotech, Inc. GA 30092.

For the patient group, the echocardiographic examination and the blood sampling for CBC and BNP assay were performed on the fourth day following blood transfusion.^22^

### Statistical analysis

The data were processed on an IBM-PC compatible computer using SPSS version 16 (SPSS Inc., Chicago, IL, USA). Continuous parametric variables were presented as means± SD while for categorical variables numbers (%) were used. Chi-square test was used for qualitative variables. The difference between 2 groups was performed by student’s t-test for parametric continuous variables and Man Whitney (U) test for non-parametric variables. Pearson correlation (r): was the test used to measure the association between two quantitative parametric variables and Spearman correlation coefficient was applied for non-parametric data. Receiver Operating Characteristic curve (ROC curve) analysis is a graph of sensitivity against 1- specificity at different cutoff points. The optimal cutoff point is that gives the highest sensitivity and specificity. Two-sided *p* value of < 0.05 was considered statistically significant.

## Results

For the patient group, their ages at diagnosis ranged from 0.5–1.5 years with a mean of 0.77± 0.24 years. The mean age of first blood transfusion was 0.69 ± 0.21 years with a range of 0.5–1 year. The mean duration of transfusion treatment was 10.1 ± 5.11 years, that of the number of the transfusions/year was 11.2 ±1.22 (median of 11 transfusions/year).

Comparison between the studied groups regarding clinical and laboratory data were represented in Table 1. The studied groups were matched regarding age, sex, the mean body weight, the mean height, and pulse rate. History of splenectomy was documented in 12 (48%) of TM patients. The patient group had a significantly lower post-transfusion Hb level with significantly higher mean yearly serum ferritin and plasma BNP levels.

The conventional echocardiography parameters were presented in Table 2. Compared to the controls, TM patients had significant higher AO, LA, LVEDD, LVESD diameters and the mitral inflow early diastolic wave velocity (E). No significant difference was found between the studied groups regarding the left ventricular EF, left ventricular FS, the mitral inflow late diastolic wave velocity (A) or the E/A ratio.

As regard to the pulsed wave TDI parameters(Table 3), TM patients had significant higher ICT and lower ET compared to the controls in all tested sites of the mitral annulus as well as in the mean values of these parameters. The mean IRT and its values at the septal and the lateral walls of the mitral annulus were significantly higher in TM compared to the controls. The mean E′ as well as its values at the lateral, anterior and inferior walls of the mitral annulus were significantly lower in TM patients compared to the controls. No significant difference was found in the S′ or A′ between the studied groups at any of the tested mitral annulus walls or in the mean values. TM patients had non-significant difference in E/E′ ratio in comparison to the controls. Abnormal E/E′ (mitral inflow E wave/E′ mitral annulus velocity) ratio (≥15) was not found in any of TM patients. According to E/E′, TM patients were classified into those with E/E′ <8 (16 patients, 64% ) and those with this ratio ≥ 8 but less than 15 (9 patients, 36%). Patients with E/E′ ≥ 8 exhibited significant higher RBCs TI, mean yearly serum ferritin and plasma BNP levels compared to those with E/E′ ratio < 8 without difference in post-transfusion Hb levels (Figure 1A and B).

Univariate analysis among TM patients revealed that serum ferritin and plasma BNP were positively correlated with each other and each of them had significant positive correlation with the mean values of E, ICT, and IRT; significant negative correlation with ET without significant correlation with E/A or E/E′ ratios (Table 4).

The data, obtained from the Roc Curve, showed that the best sensitivity of 100% and specificity of 81.9% for the plasma BNP were at cutoff point of 28.5 pg/ml in ruling out diastolic dysfunction (E/E< 8). Negative predictive value was 100 % while positive predictive value was 75%. The area under the curve was 0.86, P = 0.003 (95% CI = 0.71 – 1.01) (Figure 2A).

For the mean yearly serum ferritin, the data obtained from the Roc Curve revealed that serum ferritin level at cutoff point of 4790.5 ng/ml had the best sensitivity of 88.9% and specificity of 81.2% in ruling out diastolic dysfunction (E/E< 8). Negative predictive value was 92.9% while positive predictive value was 72, P =0.007. The area under the curve was 0.83 (95% CI = 0.64 – 1.02) (Figure 2B).

Comparison of the pulsed wave TDI mean parameters regarding the age and serum ferritin categories (age of <14 years and ≥ 14years; serum ferritin ≤2500 ng/ml and > 2500 ng/ml ) revealed that the ET was significantly lower in TM patients ≥ 14 years compared to those < 14 years. TM patients with serum ferritin >2500 ng/ml had significantly higher IRT compared to those with serum ferritin level ≤ 2500 ng/ml. There was no significant difference in the age categories regarding E/E′ ratio < 8 or ≥ 8, while there was a trend of prevalent E/E′ ratio < 8 in those with serum ferritin ≤ 2500 ng/ml (P=0.052) (Table 5).

## Discussion

Cardiac failure due to iron overload remains the most common cause of death in β-TM patients accounting for up to 71% of all deaths from this disease.^3^

Although intense iron chelation therapy can prevent and delay myocardial dysfunction, once dysfunction has become clinically evident, it is difficult to reverse.^23^

In this study, M-mode conventional echocardiography revealed a significant elevation in LV dimensions (LVEDD and LVESD) in the studied TM patients compared to the controls, which is concordant with what was reported by other investigators.^10^,^24^,^25^ This cardiac dilatation is attributed to cardiac compensation and adaptation to the chronic high-output anemic state and hypoxia.^26^

In accordance with previous studies,^20^,^27^ in our study asymptomatic TM patients had a mean values of LVEF and FS comparable to those of the control group suggesting preserved systolic function, as assessed by M-mode conventional echocardiography, till late in the disease. However, other studies reported a significantly lower LVEF in thalassemia patients in comparison with healthy age and sex-matched individuals.^28^,^29^

Echocardiographic evaluation of diastolic functions has been traditionally performed by measurement of trans-mitral flow parameters including the early (E) and late (A) diastolic filling velocities and the E/A ratio with conventional pulsed wave Doppler. The trans-mitral E wave is related to the time course of active LV relaxation that generates a pressure gradient from the left atrium through the LV inflow tract.^30^

In this study, mitral E velocity was higher in patients than in controls without a significant difference in the A velocity or E/A ratio. Similar results were reported in other studies.^14^,^24^,^29^,^31^ Absence of a significant difference in E/A ratio in TM patients compared to the controls could be due to the exclusion of patients with heart failure symptoms. It could be that the E/A alone is not sufficient to diagnose diastolic dysfunction.^29^

Nevertheless, different patterns of abnormality were documented by other researchers,^27^,^32^ who found a significant reduction in both E and A velocities in TM patients than controls without significant alteration in E/A ratio. Moreover, E/A ratio was found to be increased in thalassemia patients in the study done by Garadah et al.,^33^ denoting restrictive diastolic dysfunction. In this regard, out of our studied 25 TM patients, only 2 were found to have E/A ratio > 2 (denoting restrictive filling pattern - grade 3 diastolic dysfunction) while the others had this ratio between 1 and 2 (normal value). It has been postulated that myocardial iron deposition in some TM patients may not directly affect left ventricular contractility, but it may rather cause left ventricular myocardial restriction causing this restrictive diastolic dysfunction.^34^

In fact, LV diastolic function, as measured by conventional pulsed Doppler transmitral flow recordings, is limited by its dependence on age and loading conditions.^22^

So, elevated E velocity among our studied TM patients who were anemic compared to the controls without higher mean E/A ratio reflects an increase in the preload state due to chronic anemia.^35^ However, considering the 2 patients with E/A ratio > 2, iron deposition was the contributing factor that dominated the preload state resulting in this restrictive pattern.

Cardiac iron deposition in TM patients was found to be patchy and more in myocytes rather than the interstitium.^36^ This contributes to regional wall motion abnormalities found in these patients.^37^

Pulsed wave TDI can detect regional systolic and diastolic myocardial dysfunctions earlier than global dysfunction in thalassemia patients.^23^ It has the advantage that the measured velocities have been reported to be independent of loading conditions. 38

In the current study, pulsed wave TDI of TM patients revealed presence of systolic dysfunction in the form of significantly higher mean and regional ICT and lower ET values compared to the controls with non-significant lower regional and mean values of the systolic velocity S′. TM patients ≥ 14 years old had significant lower mean ET compared to those below 14 years without any difference in other parameters, meaning that systolic dysfunction was more evident by increasing age.

Significant reduction in the lateral annulus ejection time of TM patients (age ≤16 years) compared with healthy subjects was reported by Iarussi et al.^14^ Furthermore, Abdelmoktader and Azer 28 and Garadah et al.^33^ had reported significant lower tissue Doppler systolic velocity in the β-TM group compared to controls.

In terms of diastolic dysfunction indices, the studied TM patients had significantly lower early diastolic velocity E′ and higher IRT.

Decreased E′ is one of the earliest markers of diastolic dysfunction and is present in all stages of diastolic dysfunction.^13^ Reduced E′ velocity in TM patients was found by other researchers who attributed this to myocardial stiffness.^28^,^33^

Prolonged IRT had been previously reported in TM patients with normal systolic function.^27^,^39^ It was suggested that prolonged IRT to be the earliest sign of diastolic dysfunction in these patients and to reflect iron-induced impairment in left ventricular relaxation.^39^

Combining trans-mitral flow velocity with annular velocity (E/E′) has been proposed as a tool for assessing LV filling pressures since it is influenced by both trans-mitral driving pressure and myocardial relaxation.^40^ Because E′ velocity remains reduced and mitral E velocity increases with higher filling pressure, the ratio between trans-mitral E and E′ (E/E′ ratio) correlates well with LV filling pressure or pulmonary capillary wedge pressure (PCWP). The subjects have diastolic dysfunction when PCWP is ≥20 mm Hg if E/E′ is ≥15, a normal function if E/E′ is < 8^19^,^38^ and a suspected diastolic dysfunction having a E/E′ ratio between 8 and 15 (grey-intermediate zone).^19^,^20^

The use of E/E′ ratio has provided independent and incremental diagnostic and prognostic information in some major cardiac diseases, including heart failure.^13^ It has attracted attention for assessing LV diastolic function due to its load independent nature, its unaffection by elevated LA pressure and linear correlation with LV end diastolic pressure.^27^

E/E′ ratio has a peculiar diagnostic importance for diastolic dysfunction among TM patients.^41^

In this regard, our study results revealed that the mean E/E′ of the TM patients was higher compared to the controls yet the difference did not reach a significant level (P >0.05). None of the studied TM patients had E/E′> 15 but in 9 out of the studied 25 patients (36%), E/E′ ratio was in the gray zone, between 8 and 15, while the other 16 (64%) patients had normal E/E′ ratio of <8. Regarding E/E′ ratio, both patient categories had comparable post-transfusion Hb level with higher serum ferritin and BNP in those with a high ratio. The non-significant elevation of E/E′ ratio is in accordance with Agha et al.^31^ but not with Kremastinos et al.^20^ and Parale et al.^27^ who found that there was a significant elevation in E/E′ in the TM patients compared to the control group. This difference regarding the E/E′ ratio with the 2 mentioned studies^20^,^27^ could be related to the difference in the age range, the iron load and the use of chelation therapy. Actually, our included children were younger than those of the first study (12.0 ±5. 79 years versus, 27.2 ± 12.5 years for our patients and their patients respectively). Also all involved patients in our study were under regular chelation therapy with lower mean yearly serum ferritin level than those of the second study (4126.12±3042.80 ng/ml versus 8370.85±2660.35 ng/ml respectively) who did not receive any chelation therapy before.

It is worth mentioning that, our results indicate the absence of global LV diastolic dysfunction that is in accordance with what was reported before.^10^,^22^

So, among our non-symptomatic TM patients having a normal global systolic function by conventional echocardiography, the pulsed wave TDI had detected combined latent systolic and diastolic dysfunctions denoting the importance of this technique. By this, pulsed wave TDI detected combined systolic and diastolic dysfunctions in thalassemia patients as in previous reports.^23^,^28^

Considered as a surrogate marker for heart failure,^16^,^17^ BNP was assayed in this work. Our studied TM patients exhibited significantly higher BNP compared to the controls being higher in those with suspected diastolic dysfunction (E/E′ ≥ 8). Its level demonstrated significant correlations with systolic (positive correlation with ICT and negative correlation with ET) and diastolic ( positive correlation with both E wave and IRT) dysfunction indices, with a positive correlation trend with E/E′ ratio (P= 0.08). BNP also had good correlation with serum ferritin as an indicator of iron overload. Elevated BNP in TM patients and its association with diastolic dysfunction was documented in previous studies.^20^,^41^

Nikolidakis et al.^42^ found that BNP levels were statistically higher in the severely iron-loaded thalassemia group (with MRI T2* < 24 ms). BNP levels inversely correlated with myocardial T2 relaxation time values. The authors adopted a cut-off value of 29 pg/ml (sensitivity of 88%, specificity of 58%, negative predictive value of 94% and positive predictive value of 37%) for the identification of patients with severe myocardial hemosiderosis using magnetic resonance imaging as the comparative “gold” standard.

In accordance, our results revealed a BNP level of 28.5 pg/ml in ruling out diastolic dysfunction (E/E′< 8) and to discriminate patients with E/E′ ≥ 8 (suspected diastolic dysfunction) indicating the importance of BNP in prediction of cardiac iron load and its induced diastolic dysfunction before conventional indices of systolic function are affected.

Iron overload is the main determinant of cardiac morbidity in TM patients.^4^,^5^

Although not completely reliable, serum ferritin is the wildly accepted parameter of iron overload in clinical practice.^43^ Single ferritin measurement may be misleading and it does not reflect long-term ferritin levels or correlate with cardiac iron levels.^44^ However, the yearly trends in ferritin levels may reflect the direction of body iron loading and long term elevations in ferritin predict cardiac mortality.^45^ By this, the mean yearly serum ferritin during the last year of our enrolled TM patients, had a significant relation to pulsed wave conventional and TDI systolic and diastolic dysfunction indices. It also had significant positive correlation with BNP, the chemical marker of cardiac dysfunction. In addition it was significantly higher among patients with E/E′ ratio ≥8 than those with this ratio of <8, finding that was coupled with significant higher RBC s consumption in these patients. However, in some previous studies, no relation was found between diastolic dysfunction and serum ferritin level.^28^,^33^

Serum ferritin level of > 2500 ng/ml had been suggested to indicate increased risk of cardiac affection.^3^ The studied TM patients with serum ferritin > 2500 ng/ml had significantly higher IRT compared to those with serum ferritin level ≤ 2500 ng/ml with a trend of prevalent E/E′ ratio < 8 (normal diastolic function) in those with serum ferritin < 2500 ng/ml (P =0.052). However, using the ROC curve, serum ferritin level of 4790.5 ng/ml was the cutoff value to predict E/E′ ratio ≥ 8. In this regards, El Beshlawy et al.^46^ had reported that there was a low prevalence of myocardial siderosis as measured by cardiovascular magnetic resonance (MRI T2*) in the Egyptian TM patients in spite of very high serum ferritin and high liver iron concentration (LIC). The authors postulated that, the possibility of a genetic component for the resistance to cardiac iron loading in this population should be considered. This could reflect genetic susceptibility to cardiac iron toxicity in different populations and needs further large-scale studies.

In summary, cardiac dysfunction is a common morbidity among iron-loaded TM patients despite regular chelation therapy. In this study cardiac non-symptomatic TM patients with normal global systolic functions by conventional Echo-Doppler measurements demonstrated abnormal left ventricular systolic and diastolic indices using the pulsed-wave TDI study which were correlated with plasma BNP and serum ferritin. This confirms the importance of this diagnostic modality in TM patients especially in developing countries where wide use of cardiovascular magnetic resonance (MRI T2*) is limited by its expensiveness.^23^ BNP cut-off value for prediction of diastolic dysfunction (E/E′ ≥8) was more or less equal to the previously documented one for prediction of MRI T2* < 24 ms (28.5 pg/ml vs 29 pg/ml) and this raise the concern about the importance of this simple un-expensive test in iron-loaded TM patients.

## Conclusions

Asymptomatic TM children under regular chelation therapy may have latent diastolic and or systolic dysfunctions that could not be detected by conventional echocardiography but could be highlighted by TDI. Hence, application of pulsed-wave TDI in these patients is appropriate. Integrated use of echocardiography, pulsed-wave TDI and BNP level for an accurate assessment of cardiac functions is highly recommended to help identifying subjects at risk and facilitates early intervention.

## Figures and Tables

**Figure 1a:**
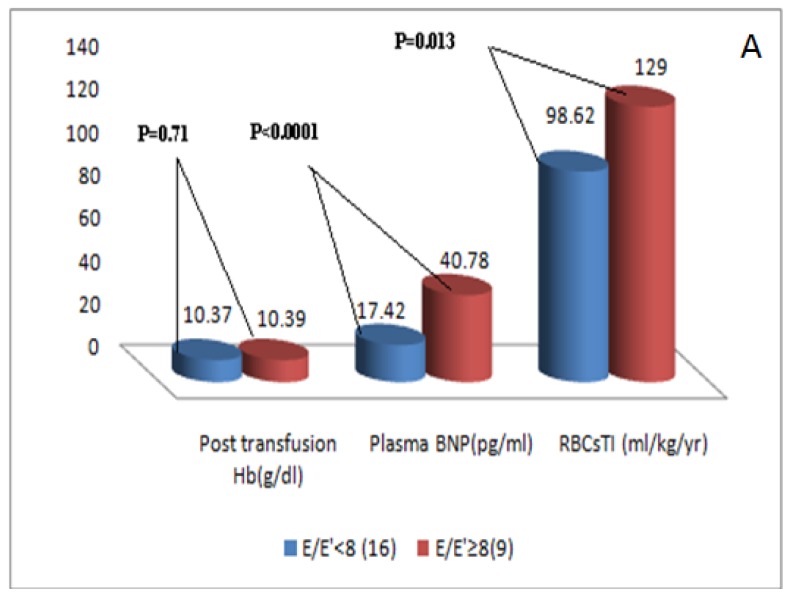
Comparison of post transfusion Hb ( g/dl), plasma BNP (pg/ml) and RBCs TI (ml/kg/year) in TM patients with E/E′ ratio ≥8 compared to patients with E/E′ <8.

**Figure 1b:**
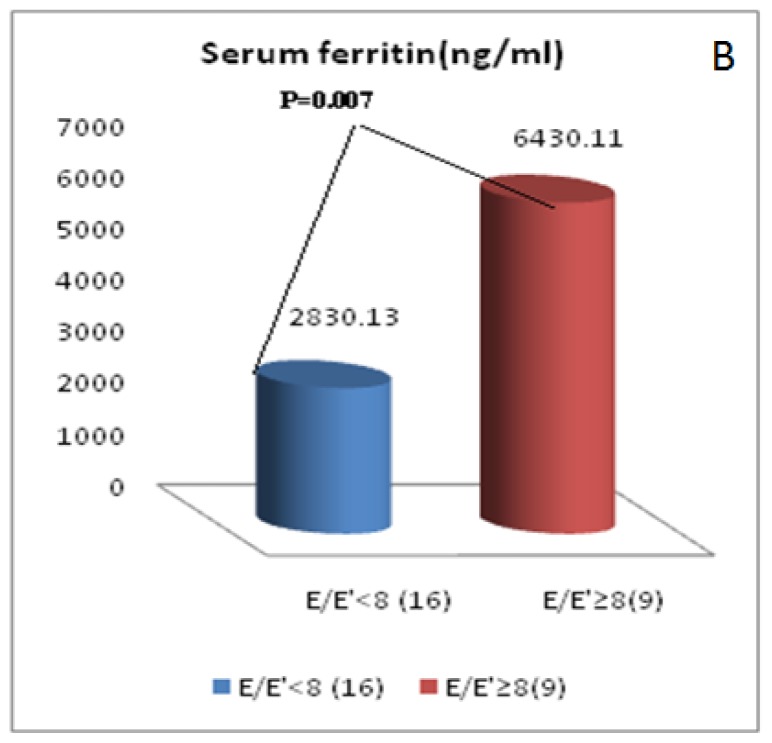
Comparison of mean yearly serum ferritin ( ng/ml) in TM patients with E/E′ ratio ≥8 compared to patients with E/E′ <8.

**Figure 2A:**
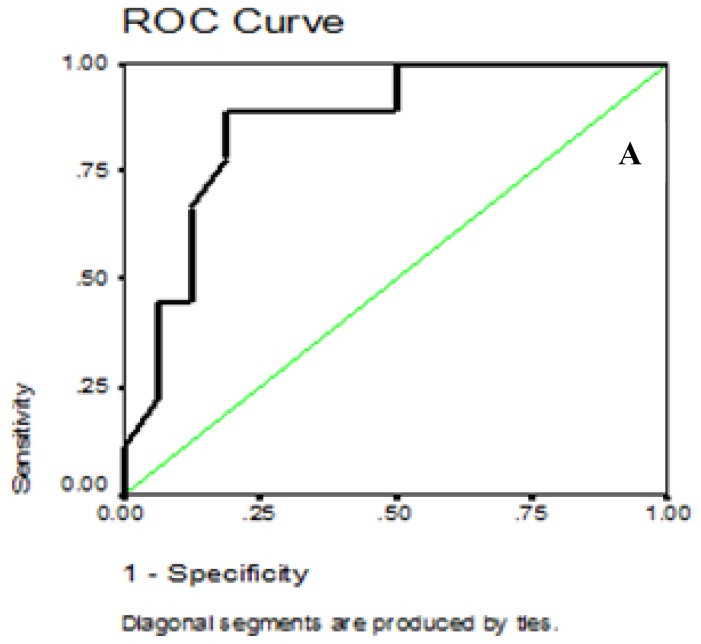
Receiver operator characteristic curve of BNP values for the identification of patients with E/E′ ≥8.

**Figure 2B:**
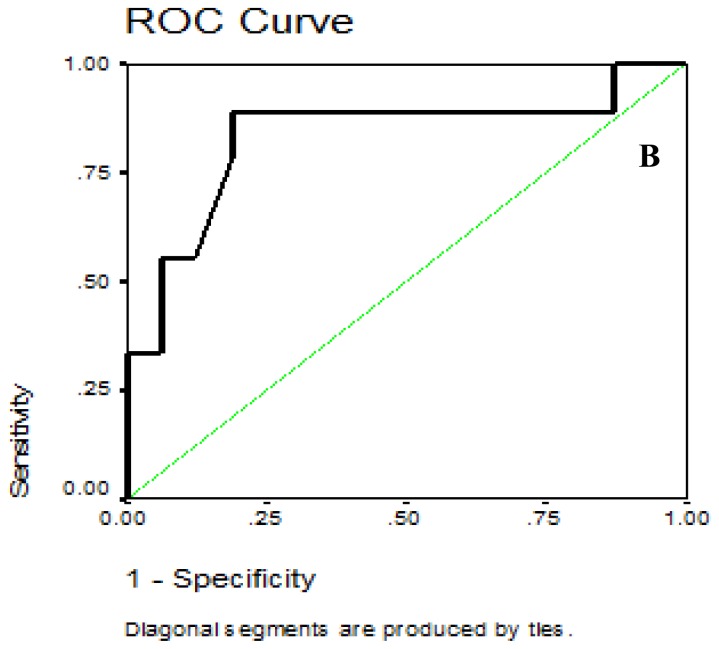
Receiver operator characteristic curve of serum ferritin values for the identification of patients with E/E′ ≥8.

**Table 1:** Comparison of the clinical and laboratory parameters between TM patients and controls**.**

Variables	Patients ( 25)	Controls ( 20)	P value
Age (years)	12.0 ±5. 79	10.9± 4.86	0.56
Gender, males, n(%)	14(56)	10(50)	1
Body weight (kg)	27.88±10.68	40.87±21.6	
< 5^th^ percentile, n(%)	6(24)	0(0)	0.19
Normal percentile range, n (%)	19(76)	20(100)	**0.019**
Height (cm)	129.28±21.63	137.53±31.53	0.23
< 5^th^ percentile, n (%)	9(36)	0(0)	
Normal percentile range, n(%)	16(64)	20(100)	**0.003**
Pulse (beat/min)	90.32±6.24	83.2±11.22	0.064
Splenectomy, n (%)	12(48%)	-	-
RBCs TI(ml/kg/year)	109.75±30.39	-	-
Chelation, n (%)			
DFO	16(64)	-	-
Deferasirox	4(16)		
DFO+Deferasirox	5(20)		
Pre transfusion Hb(g/dl)	7.28±1.03	12.2 ±0.58	**<0.0001**
Post transfusion Hb(g/dl)	10.38±0.41	12.2±0.58	**<0.0001**
Serum ferritin (ng/ml)	4126.12±3042.80	124.50± 22.81	**<0.0001**
Median	4408	122	
Range	825–10200	96–170	
BNP (pg/ml)	29.28±14.30	8.80 ± 1.81	**<0.0001**

Bold numerical values indicate significance.

**Table 2:** Comparison of conventional echocardiographic measures between TM patients and the controls.

Variables	Patients (25)	Controls ( 20)	P value
AO(mm)	22.92±4.22	19.40±3.61	**0.02**
LA(mm)	30.40±5.84	23.60±8.0	**0.01**
LVEDD(mm)	46.48±8.38	33.30±3.92	**<0.0001**
LVESD(mm)	29.16±5.83	20.80±2.48	**<0.0001**
EF(%)	66.24±5.57	69.50±3.34	0.11
FS (%)	37.48±6.65	38.20±2.44	0.31
E(cm/sec)	112.60±19.91	91.0±15.22	**0.001**
A(cm/sec)	75.20±22.65	69.60±14.07	0.56
E/A ratio	1.54±0.34	1.36±0.29	0.28

AO: aortic diameter; LA: left atrium diameter; LVEDD: left ventricular end diastolic diameter; LVESD: left ventricular end systolic diameter; EF: ejection fraction; FS: fraction shortening; E: mitral inflow early diastolic wave; A: mitral inflow late diastolic wave; E/A: mitral inflow early to late diastolic wave ratio. Bold numerical values indicate significance.

**Table 3:** Comparison of Pulsed wave TDI measures between TM patients and the controls.

	Septal wall	Lateral wall	Anterior wall	Inferior wall	The mean
S′ (cm/s)					
Patients	7.90±0.87	8.92±2.21	7.90 ±0.88	8.84±1.34	8.28±0.9
Controls	8.56±1.98	9.10±1.37	8.04±2.17	8.20 ±1.62	8.6±1.26
P value	0.53	0.59	0.54	0.15	0.47
E′ (cm/s)					
Patients	11.50±2.22	12.90±1.85	11.0±2.36	12.90 ±2.77	12.07 ±2.06
Controls	13.16±3.92	16.84±3.32	14.40±2.52	15.48±3.15	15.04±2.65
P value	0.14	**0.001**	**0.002**	**0.04**	**0.003**
A′ (cm/s)					
Patients	7.90 ±1.45	7.70±0.67	7.10±1.20	7.80 ±1.40	7.45 ±0.92
Controls	7.92±2.63	7.76±2.52	7.4±1.19	9.16±2.09	8.2±2.02
P value	0.94	0.61	0.69	0.12	0.48
ICT(ms)					
Patients	66.88±8.98	69.96±11.02	67.16±14.40	64.6±17.79	67.15±10.89
Controls	45.0 ±8.26	58.20±29.76	41.30±5.12	45.90 ±9.53	47.85±8.97
P value	**<0.0001**	**0.001**	**<0.0001**	**0.001**	**<0.0001**
ET(ms)					
Patients	231.70±17.99	228.20±30.20	231.60±29.60	225.40 ±20.95	229.22±23.17
Controls	250.96±24.34	252.16±23.58	254.88±23.42	253.08±24.24	252.77±22.25
P value	**0.02**	**0.03**	**0.04**	**0.008**	**0.02**
IRT(ms)					
Patients	59.60±10.11	53.0±10.84	56.72±10.15	56.08±12.64	56.28±8.35
Controls	46.90 ±8.05	45.40±6.57	51.90±10.49	50.60 ±9.51	48.7±4.18
P value	**0.001**	**0.04**	0.14	0.49	**0.008**
E/E′					
Patients					8.0±2.09
Controls					7.42 ±0.3
P value					0.77

S′: mitral annulus systolic velocity; E′: mitral annulus early diastolic velocity; A′: mitral annulus late diastolic velocity; ICT: isometric contraction time; ET: ejection time; IRT: isovolumic relaxation time; E/E′: mitral inflow early diastolic wave E/mitral annulus early diastolic velocity E′ ratio. Bold numerical values indicate significance

**Table 4:** Correlations between BNP and serum ferritin with different conventional echo cardiography and TDI parameters.

	BNP (pg/ml)		Serum ferritin (ng/ml)	

	Correlation coefficient (r)	P value	Correlation coefficient (r)	P value
Serum Ferritin (ng/ml)	+ 0.63	**<0.0001**		
E (cm/sec)	+ 0.62	**<0.0001**	+ 0.59	**<0.0001**
E/A	+ 0.29	0.09	+ 0.12	0.48
ICT(ms)	+ 0.48	**0.003**	+ 0.57	**<0.0001**
ET (ms)	− 0.68	**<0.0001**	− 0.44	**0.008**
IRT(ms)	+ 0.45	**0.007**	+ 0.55	**0.001**
E/E′	+ 0.30	0.08	+ 0.25	0.14

E: mitral inflow early diastolic wave; A: mitral inflow late diastolic wave; E/A: mitral inflow early to late diastolic wave ratio; ICT: isometric contraction time; ET: ejection time; IRT: isovolumic relaxation time; E/E′: mitral inflow early diastolic wave E/mitral annulus early diastolic velocity E′ ratio. Bold numerical values indicate significance.

**Table 5:** Comparison of Pulsed wave TDI mean measures among TM patients regarding the age and serum ferritin categories.

	Age < 14 years (14)	Age ≥14 years (11)	P value	Serum ferritin ≤2500 (ng/ml) (9)	Serum ferritin >2500 (ng/ml) (16)	P value
S′ (cm/s)	8.79±0.99	8.45±1.45	0.5	8.7±1.4	8.42±1.00	0.6
E′ (cm/s)	15.54±2.68	14.41±2.61	0.3	14.51±2.38	15.97±2.99	0.19
A′ (cm/s)	8.54±2.34	7.77±1.52	0.36	9.00±2.42	7.75±1.67	0.14
ICT(ms)	67.66±9.81	67.32±11.37	0.94	65.69±10.73	68.53±10.25	0.52
ET(ms)	268.02±15.14	237.07±25.33	**0.002**	258.16±17.86	237.42±34.04	0.09
IRT(ms)	54.25±7.99	58.79±8.33	0.18	51.78±7.4	58.76±7.88	**0.04**
E/E′	7.41±1.41	8.76±2.61	0.11	7.12±0.74	8.5±2.45	0.14
E/E′						
<8	11(78.6)	5(45.5)		8(88.9)	8(50)	
≥ 8	3(21.4)	6(54.5)	0.087	1(11.1)	8(50)	0.052

S′: mitral annulus systolic velocity; E′: mitral annulus early diastolic velocity; A′: mitral annulus late diastolic velocity; ICT: isometric contraction time; ET: ejection time; IRT: isovolumic relaxation time; E/E′: mitral inflow early diastolic wave E/mitral annulus early diastolic velocity E′ ratio. Bold numerical values indicate significance.
